# Supplementary material for: Do restrained eaters show increased BMI, food craving and disinhibited eating? A comparison of the Restraint Scale and the Restrained Eating scale of the Dutch Eating Behaviour Questionnaire
Source: R Soc Open Sci. 2019 Jun 12;6(6):190174. doi: 10.1098/rsos.190174 (PMC6599805; doi:10.1098/rsos.190174)
Supplement: Supplementary Information: RQC [file rsos190174supp1.docx]

**Supplementary Information:**

Do restrained eaters show increased BMI, food craving, and disinhibited eating?

A comparison of the Restraint Scale and the Restrained Eating scale of the Dutch Eating Behaviour Questionnaire

**Table S.1.** Internal consistency for the scales used.

|  | Cronbachs alpha (α) | | | |
| --- | --- | --- | --- | --- |
|  | Sample 1  (min. N=1265) | Sample 2  (min. N=204) | Sample 3  (min. N=202) | Total  (min. N=1514) |
| RS | 0.82 | 0.85 | 0.85 | 0.83 |
| RSCD | 0.79 | 0.83 | 0.83 | 0.8 |
| RSWF | 0.74 | 0.75 | 0.74 | 0.74 |
| DEBQRE | 0.91 | 0.94 | 0.93 | 0.92 |
| DEBQEE | 0.85 | 0.86 |  | 0.85 |
| ACQC | 0.93 | 0.91 |  | 0.93 |
| TFEQD |  |  | 0.82 |  |

Note. RS= Restraint Scale; RSCD= concern for dieting subscale of the RS; RSWF= weight fluctuation scale of the RS; DEBQRE= Dutch Eating Behaviour Questionnaire Restrained Eating scale; DEBQEE= Dutch Eating Behaviour Questionnaire External Eating scale; ACQC= Attitudes to Chocolate Questionnaire Craving scale; TFEQD = Three Factor Eating Questionnaire Disinhibited Eating scale

***Factor Structure***

Kaiser-Meyer-Olkin (KMO; Kaiser, 1974) measures of sampling adequacy indicated that factor extraction could be performed for all samples (RS: all >0.85; DEBQRE: all >0.92). For the RS, extraction revealed two factors with eigenvalues greater than 1 for all samples (see Table S.2). Factors were consistent with the RSCD and RSWF subscales, although some items loaded onto both factors (factor loadings greater than 0.4 are shown in Table S.2.). These factors combined explained more variance than when a forced extraction of one factor was used. One factor was revealed for the DEBQRE with an eigenvalue of >1.

**Table S.2.** Factor extraction for the RS and DEBQRE.

|  | Sample 1  (min. N=1265) | | | Sample 2  (min. N=204) | | | Sample 3  (min. N=202) | | | Total  (min. N=1671) | | |
| --- | --- | --- | --- | --- | --- | --- | --- | --- | --- | --- | --- | --- |
|  | No. principal components | Component | Variance explained (%) | No. principal components | Component | Variance explained (%) | No. principal components | Component | Variance explained (%) | No. principal components | Component | Variance explained (%) |
| RS | 2 | 1 | 29.25 | 2 | 1 | 35.12 | 2 | 1 | 32.93 | 2 | 1 | 30.47 |
|  |  | 2 | 24.27 |  | 2 | 22.36 |  | 2 | 24.26 |  | 2 | 23.87 |
| DEBQRE | 1 | 1 | 55.23 | 1 | 1 | 64.26 | 1 | 1 | 60.48 | 1 | 1 | 57.03 |

Note. RS= Restraint Scale; DEBQRE= Dutch Eating Behaviour Questionnaire Restrained Eating scale.

**Table S.3.** Factor loadings for the RS. Only loadings >0.4 are depicted.

|  | Sample 1  (min. N=1265) | | Sample 2  (min. N=204) | | Sample 3  (min. N=202) | | Total  (min. N=1671) | |
| --- | --- | --- | --- | --- | --- | --- | --- | --- |
|  | Factor 1 (RSCD) | Factor 2 (RSWF) | Factor 1 (RSCD) | Factor 2 (RSWF) | Factor 1 (RSCD) | Factor 2 (RSWF) | Factor 1 (RSCD) | Factor 2 (RSWF) |
| 1. How often dieting? | 0.66 | 0.43 | 0.78 |  | 0.75 |  | 0.68 |  |
| 2. Max weight lost 1m? |  | 0.68 | 0.42 | 0.52 |  | 0.62 |  | 0.66 |
| 3. Max weight gain 1wk? |  | 0.83 |  | 0.86 |  | 0.86 |  | 0.84 |
| 4. Weight fluctuate 1wk? |  | 0.77 |  | 0.81 |  | 0.78 |  | 0.78 |
| 5. Effect of 5lb change? | 0.73 |  | 0.76 |  | 0.73 |  | 0.73 |  |
| 6. Splurge alone? | 0.52 |  | 0.44 |  | 0.51 |  | 0.52 |  |
| 7. Thought to food? | 0.67 |  | 0.71 |  | 0.70 |  | 0.68 |  |
| 8. Feelings of guilt? | 0.77 |  | 0.78 |  | 0.78 |  | 0.77 |  |
| 9. Conscious of eating? | 0.68 |  | 0.74 |  | 0.77 |  | 0.70 |  |
| 10. Max. overweight? |  | 0.64 | 0.52 | 0.57 | 0.43 | 0.62 | 0.41 | 0.63 |

Note. Loadings underlined are the highest loadings across both factors for each sample. RS= Restraint Scale; RSCD= concern for dieting subscale of the RS; RSWF= weight fluctuation scale of the RS.

***Demographic Differences***

Due to the large differences in restraint scores between genders, correlations with age were split according to gender^[[1]](#footnote-1)^ (see Table S.4.). Female respondents showed a significant positive correlation between age and scores on the RS (*r*=0.08, *p*=0.004) and a significant positive correlation between age and RSWF (*r*=0.14, *p*<0.001). However, whereas women showed no statistically significant relationships between either RSCD (*r*=0.01, *p*=0.63) or DEBQRE (*r*=0.003, *p*=0.91) and age, men did show a significant positive relationship between both RSCD and age (*r*=0.26, *p*<0.001) and DEBQRE and age (*r*=0.17, *p*=0.003). Men also showed significant positive correlations between age and both RS (*r*=0.27, *p*<0.001) and RSWF *(r*=0.19, *p*=0.001). The difference in correlations between genders for RSCD (*z*=3.94, *p*<0.001), DEBQRE (*z*=2.64, *p*=0.008) and RS and age (*z*=3.06, *p=*0.002) were all statistically significant but the difference in correlations between genders for RSWF and age was not (*z*=0.75, *p*=0.45).

**Table S.4.** Means and total scores for restrained eating collapsed across samples 1-3 according to gender (SE within parentheses).

|  | Samples 1-3 | | | | |
| --- | --- | --- | --- | --- | --- |
|  |  | Females  (min. N=1383) | Males  (min. N=301) | *p* | *d* |
| RS | Mean | 1.37 (0.02) | 1.03 (0.03) | <0.001 | 0.6 |
|  | Total | 13.63 (0.16) | 10.27 (0.3) |  |  |
| RSCD | Mean | 1.46 (0.02) | 1.09 (0.03) | <0.001 | 0.63 |
|  | Total | 8.73 (0.1) | 6.52 (0.18) |  |  |
| RSWF | Mean | 1.25 (0.02) | 0.95 (0.04) | <0.001 | 0.39 |
|  | Total | 4.93 (0.08) | 3.77 (0.17) |  |  |
| DEBQRE | Mean | 2.74 (0.02) | 2.13 (0.04) | <0.001 | 0.76 |
|  | Total | 27.37 (0.23) | 21.33 (0.43) |  |  |

Note. *p* values have not been corrected for multiple comparisons. RS= Restraint Scale; RSCD= concern for dieting subscale of the RS; RSWF= weight fluctuation scale of the RS; DEBQRE= DEBQ Restrained Eating scale

**Table S.5.** Correlations between restrained eating measures and measures of general food craving – trait version (G-FCQ-T; Cepeda-Benito et al*.*, 2000; Nijs et al*.*, 2007) for sample 4 who scored highly on the RS (15+) and ACQC (10+) and participated in one of the experimental studies in Adams et al. (2017).

|  | Sample 4  (min. N=213) | | | | |
| --- | --- | --- | --- | --- | --- |
|  | FCQ-PWF | FCQ-LoC | FCQ-POE | FCQ-EC | FCQ-total |
| RS | 0.22** | 0.25*** | 0.01 | 0.20** | 0.22** |
| RSCD | 0.24*** | 0.17* | -0.04 | 0.19** | 0.18** |
| RSWF | 0.003 | 0.13~ | 0.07 | 0.03 | 0.07 |
| DEBQRE | -0.06 | -0.04 | -0.10 | -0.01 | -0.06 |

****p*<0.001 ***p*<0.01 **p*<0.05 ~*p*<0.1

Note. *p* values have not been corrected for multiple comparisons. RS= Restraint Scale; RSCD= concern for dieting subscale of the RS; RSWF= weight fluctuation scale of the RS; DEBQRE= Dutch Eating Behaviour Questionnaire Restrained Eating scale; FCQ-PWF= preoccupation with food scale of the G-FCQ-T; FCQ-LoC= loss of control scale of the G-FCQ-T; FCQ-POE= positive outcome expectancy scale of the G-FCQ-T; FCQ-EC= emotional craving scale of G-FCQ-T; FCQ-total= total score for the G-FCQ-T

1. Across both genders, correlations revealed a small but statistically significant positive association between age and RS (*r*=0.08, *p*=0.001). This was due to a small, positive correlation between age and the weight fluctuation subscale (*r*=0.13, *p*<0.001); the correlation between age and concern for dieting was not statistically significant (*r*=0.03, *p*=0.27), nor was the correlation between age and DEBQRE (*r*= -0.001, *p*=0.96). [↑](#footnote-ref-1)
